# Robust biomimetic MOF featuring a negative pocket for precise recognition of uranyl, enabling ultrahigh U/V selectivity and rapid uranium extraction from seawater†

**DOI:** 10.1039/d5sc02966j

**Published:** 2025-06-20

**Authors:** Anni Ye, Yuxuan Liu, Lele Gong, Xianqing Xie, Feng Luo

**Affiliations:** a National Key Laboratory of Uranium Resources Exploration-Mining and Nuclear Remote Sensing, East China University of Technology Nanchang 330013 China ecitluofeng@163.com; b State Key Laboratory of NBC Protection for Civilian Beijing 100191 China; c National Engineering Research Center for Carbonhydrate Synthesis, Jiangxi Normal University Nanchang 330027 China

## Abstract

Low U/V selectivity and long extraction time remain the major obstacles in the field of uranium extraction from seawater, as both can largely reduce the uranium extraction ability of an adsorbent. We present a robust biomimetic metal–organic framework (MOF) material, designated ECUT-74, engineered through innovative incorporation of helical rod-like secondary building units. This structural design introduces a protein-mimicking uranyl-binding motif that enables precise recognition of uranyl, leading to a benchmark U/V selectivity of 10^4^. When applied to real seawater (3.3 μg L^−1^), ECUT-74 demonstrates an ultrafast uranium extraction with a record-making extraction ability of 16 mg g^−1^ in just one day and 22.6 mg g^−1^ in five days, along with an extremely low uptake for other metal ions (less than 2 mg g^−1^). The robust nature of this MOF allows for a good reusability for uranium extraction from seawater. All these merits strongly suggest the superior application of ECUT-74 for uranium extraction from seawater.

## Abstract 2

We report here a MOF featuring negatively charged interhelical pockets in a three-dimensional channel, showing a remarkable U/V selectivity and a uranium uptake capacity of 16 mg g^−1^ in one day from seawater.

## Graphical abstract

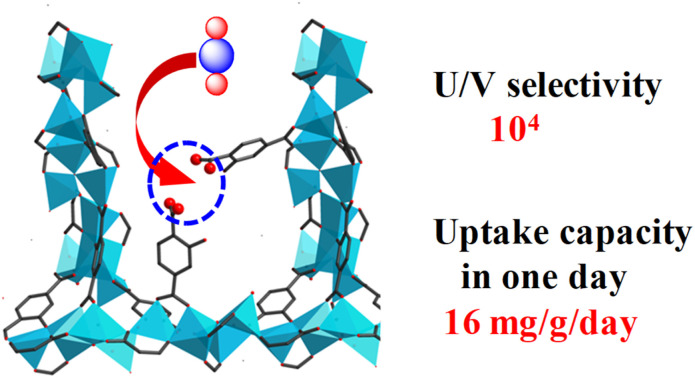

## 1. Introduction

Nuclear power, as a high-energy-density energy carrier, plays a pivotal role in addressing global energy security challenges and mitigating carbon emissions.^1–3^ However, the finite nature of uranium, the primary nuclear fuel, raises significant sustainability concerns.^4^ Current terrestrial uranium reserves are estimated at around 7.6 million metric tons, which would support approximately 80 more years of continued nuclear energy production at current rates of technology and consumption.^5^ This reality underscores the imperative to develop alternative uranium sources for long-term energy security.^6^ Seawater presents a promising solution, constituting 99.8% of Earth's liquid water inventory and containing an estimated 4.5 billion metric tons of dissolved uranium—a resource abundance sufficient to sustain nuclear power generation for millennia.^7,8^ Despite these advantages, efficient uranium recovery from seawater remains a formidable challenge, constrained by four critical factors:^9–11^ (1) uranium exists at trace levels (∼3.3 μg L^−1^) in seawater, requiring highly sensitive extraction technologies; (2) dominant cations (Na^+^, Mg^2+^, Ca^2+^) present at millimolar concentrations severely complicate selective adsorption processes; (3) significant interference from comparable ions such VO^2+^ leads to serious coadsorption and then a large reduction in the extraction ability of materials; (4) microbial colonization on extraction materials leads to rapid performance deterioration and operational instability.^12^

Given the prevalence of uranium in seawater as the complex anionic species [UO_2_(CO_3_)_3_]^4−^, the incorporation of uranyl-binding motifs in adsorbent materials becomes crucial for efficient uranium recovery.^13,14^ This functionalization enables preferential adsorption through a ligand substitution mechanism, where the uranyl-binding groups competitively displace carbonate ions (CO_3_^2−^) from [UO_2_(CO_3_)_3_]^4−^ during ion exchange processes.^15–17^ Among various functional groups, carboxyl (–COOH), sulfonic acid (–SO_3_H), and amidoxime have emerged as particularly effective ligands in this context. However, the adsorption efficiency is fundamentally governed by two critical factors: the geometric compatibility between the ligand's coordination environment and the uranyl ion's binding site and the microstructural characteristics of the adsorbent matrix. These parameters collectively determine the kinetics of uranyl adsorption.^18–33^ Despite some materials demonstrating exceptional adsorption capacities,^34–44^ for instance MIGPAF-13 (ref. 25) reaching 16 mg g^−1^ and i-MZIF90(50)^35^ achieving 28.2 mg g^−1^, their practical application remains limited due to excessively prolonged extraction times. Notably, MIGPAF-13 needs 56 days, while i-MZIF90(50) needs 25 days. PPH-OP^36^ requires 21 days for 7.12 mg g^−1^ adsorption. AO-PIM-1 (ref. 37) exhibits 9.03 mg g^−1^ capacity after 28 days. Such a prolonged uranium extraction period will pose a critical risk of severe biofouling, which not only compromises adsorption kinetics through surface occlusion but also induces mechanical degradation and structural collapse of the adsorbent matrix. This multifaceted degradation mechanism ultimately undermines operational stability and renders prolonged uranium recovery from seawater economically unviable.

**Scheme 1:**
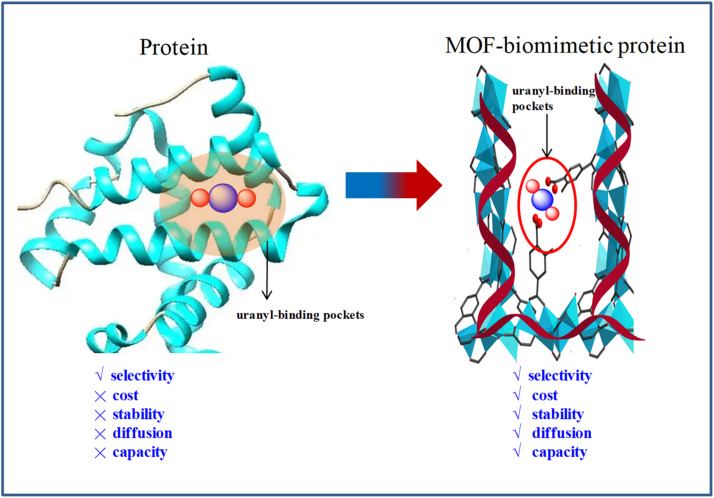
A comparison between protein and MOF-biomimetic protein in uranyl extraction from seawater.

Proteins have been considered as potential candidates for uranium extraction from seawater due to their unique ability to form negatively charged uranyl-binding pockets through specific amino acid interactions, enabling precise ligand recognition (Scheme 1).^45^ However, their inherent hydrophobic interior and compact macromolecular structure significantly impede uranium diffusion pathways within the protein matrix, thereby restricting adsorption kinetics. Furthermore, practical implementation of protein-based systems is confronted with challenges related to operational stability in seawater, as well as scalability concerns associated with recombinant protein production and purification costs. The secondary building units (SBUs) in proteins are structurally characterized by α-helical segments enriched with carboxylate groups (–COOH). Through precise arrangement of these helical motifs, proteins achieve the formation of negatively charged uranyl-binding pockets *via* coordinated carboxylate networking. Notably, such structural design principles can be systematically replicated in metal–organic framework (MOF)-inspired biomimetic architectures by engineering analogous α-helical SBUs with optimized carboxylate density and spatial configuration (Scheme 1), and we can potentially construct artificial uranyl-binding pockets that maintain both high selectivity and enhanced adsorption capacity for uranyl ions, as well as the porosity to meet the requirements of uranium diffusion pathways.^46^

To this end, we report a biomimetic Zn(ii)-based MOF, designated as ECUT-74, engineered through replication of the helical SBUs in proteins. This material demonstrates exceptional structural characteristics including: (i) a three-dimensional helical network architecture with free-standing carboxylic acid group (–COOH) as primary coordination sites, (ii) ultrahigh porosity with Brunauer–Emmett–Teller (BET) surface areas of 1150 m^2^ g^−1^, and (iii) superior chemical stability in extreme pH environments (pH 1–9, 24 h immersion test). The precisely arranged SBU motifs in ECUT-74 form a geometrically optimized uranyl-binding pocket that exactly matches one uranyl ion in the coordination surrounding. This biomimetic design enables ultrafast uranium extraction from seawater with an extraction ability of 16 mg g^−1^ in one day, along with ultrahigh selectivity towards uranium over other competing cations (such as Fe, Zn, Cu and V) and good recyclability, suggesting its superior application in quickly obtaining uranium resources from seawater.

## 2. Experimental section

### 2.1 Synthesis of ECUT-74

A mixture of 2-hydroxyterephthalic acid (36.4 mg, 0.2 mmol), 1,4-diazabicyclo[2.2.2]octane (16.8 mg, 0.15 mmol), Zn(NO_3_)_2_·6H_2_O (148.5 mg, 0.5 mmol) in 2.5 mL DMF and 0.7 mL glycol is sealed into a 25 mL reaction kettle and heated at 130 °C for two days. Upon completion of the reaction, the reaction mixture is cooled to room temperature, and the resulting crystal products are cleaned three times with DMF (2 mL) and three times with methanol (2 mL). The yield is 50.5 mg, which is about 86% based on 2-hydroxyterephthalic acid.

### 2.2 Chemical stability test

The chemical stability test of ECUT-74 is carried out by soaking 100 mg ECUT0-74 in aqueous solution with pH = 1 or 9 for 24 h. Then, the samples are filtered using a 0.24 μm filter and washed with water (50 mL) three times. PXRD, IR, solid-state ^13^C MAS NMR, TG, N_2_ adsorption, and SEM-EDS are used to investigate the chemical stability of ECUT-74.

### 2.3 Characterization of material

Uranium has mild radioactivity; therefore, it advisable to conduct related experiments under appropriate safety measures. UO_2_(NO_3_)_2_·6H_2_O (99%), the raw materials for synthesizing the MOF and the organic solvents (99%) were purchased from Aladdin Biochemical Technology Co. Ltd. All these reagents were used as received without further purification. Powder X-ray diffraction was conducted with a Bruker AXSD8 Discover powder diffractometer at 40 kV, 40 mA using Cu K*λ* (*λ* = 1.5406 Å). The simulated powder patterns were calculated by Mercury 1.4. Infrared (IR) spectra were obtained with a Bruker VERTEX70 spectrometer in the 600–2000 cm^−1^ region. The gas adsorption isotherms were recorded with a Belsorp-max. Ultrahigh-purity-grade (>99.999%) N_2_ gases were used during the adsorption measurement. The analyses of concentrations of U ions in solution were carried out with a ThermoFisher iCap7600 ICP-OES or ICP-MS-7200. X-ray photoelectron spectra (XPS) were collected with a Thermo Scientific ESCALAB 250 Xi spectrometer. Scanning electron microscopy (SEM) images were recorded with a Hitachi SU 8100 scanning electron microscope. Solid-state NMR experiments were performed using a Bruker Advance III HD solid-state NMR spectrometer (600 MHz).

### 2.4 Uranium extraction experiment

For initial U concentrations of 12.4 and 1.73 mg L^−1^ at a pH of 5, we used 5 mg ECUT-74 to carry out uranium extraction experiments. 50 mL uranium solution was used. When the set extraction time (5–1440 minutes) was finished, the solution was filtered using a 0.24 μm filter. The residual uranium concentration was determined by ICP-OES and ICP-MS. Triplicate tests were used to calculate the error bars.

For initial U concentrations of 119.0, 10.0, and 3.86 μg L^−1^ at a pH of 5, we also used 5 mg ECUT-74 to carry out uranium extraction experiments. 50 mL uranium solution was used. After extracting for 24 h, the solution was filtered using a 0.24 μm filter. The residual uranium concentration was determined by ICP-MS. Triplicate tests were used to calculate the error bars.

In the adsorption isotherm measurements, initial U concentrations of 12.4, 50.7, 114.5, 156.8, and 208.6 mg L^−1^ were used, along with 5 mg ECUT-74 at pH = 5. 50 mL uranium solution was used. After extracting for 24 h, the solution was filtered using a 0.24 μm filter. The residual uranium concentration was determined by ICP-OES. Triplicate tests were used to calculate the error bars.

The reusability experiments were conducted as follows. First, we used 5 mg ECUT-74 to carry out uranium extraction. 50 mL of 10 mg L^−1^ U solution at a pH value of 5 was used. After extracting for 60 min, the solution was filtered using a 0.24 μm filter. The residual uranium concentration was determined using ICP-OES. Then the filtered solid was treated with 0.1 M HNO_3_ for 24 h. The uranium concentration resulting from using 0.1 M HNO_3_ as an eluent was determined by ICP-OES. Additionally, the ECUT-74 was regenerated and recovered by filtration using a 0.24 μm filter and further natural drying at room temperature for 24 hours. Then, the regenerated ECUT-74 was used to conduct a second uranium extraction experiment. After that, an adsorption–desorption cycle was finished. Triplicate tests were used to calculate the error bars.

The *K*_d_ value was calculated from eqn (1):1
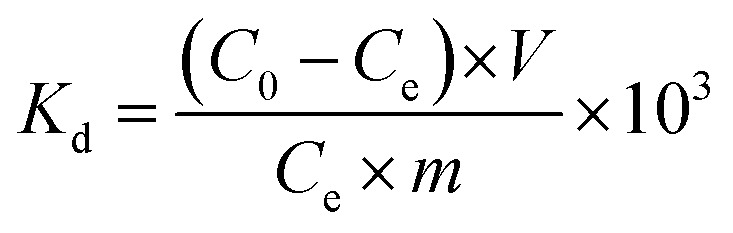
where *C*_0_ (mg L^−1^) and *C*_e_ (mg L^−1^) are the initial concentration and equilibrium concentration of uranium in the solutions, respectively; *V* (mL) is the volume of testing solution; and *m* (mg) is the amount of adsorbent.

### 2.5 Uranium selectivity experiment

In the selective adsorption experiments, we used the binary ion system with an initial concentration of 1 mg L^−1^ for uranium ions and 100 mg L^−1^ for other ions (Na, Mg, K, and V) at a pH value of 8, while we used a mixed ion system with an initial concentration of 1 mg L^−1^ for uranium ions and other ions (Na, K, Mg, Ca, Cu, Ni, Mn, Zn, Sr, Pb, V, and U) at a pH value of 8. We used 5 mg ECUT-74 to carry out the uranium selectivity experiments. 50 mL mixed solution was used. After extracting for 24 h, the solution was filtered using a 0.24 μm filter. The residual uranium concentration was determined by ICP-MS, while the residual concentration of the other ions was determined by ICP-OES. Triplicate tests were used to calculate the error bars. Similarly, *S*_U/M_ was calculated from eqn (2):2
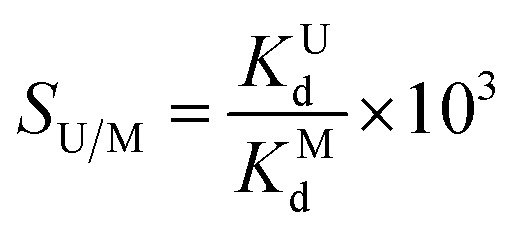


### 2.6 Extraction of uranium from seawater

Seawater (100 L) at pH = 8 collected from the South China Sea, Zhuhai, was not filtered and directly used for the uranium extraction experiments. First, we built a column cyclic adsorption system, with which the seawater flowed through the adsorbent at a speed of 0.66 L min^−1^. The dose of ECUT-74 was 10 mg. The initial concentration of uranium and other crucial competing cations (Zn, Cu, Fe, and V) in seawater was determined by ICP-MS, giving 3.3 μg L^−1^ for uranium. When the set extraction time (1–5 days) was finished, corresponding seawater with a volume of 10 mL was taken to determine the residual concentration of uranium and other metals (Zn, Cu, Fe, and V) in the seawater by ICP-MS. Triplicate tests were used to calculate the error bars.

Reusability experiments were conducted as follows. First, after finishing the adsorption experiment, for the desorption process, we used 0.1 M HNO_3_ (1 L) as an eluent to flow through the adsorbent at a speed of 0.05 L min^−1^ for 24 h. After that, deionized water (5 L) was used to flow through the adsorbent at a speed of 0.1 L min^−1^ for 2 h, which was repeated three times to achieve a full recovery of adsorbent. Subsequently, we repeated the adsorption experiment. After that, an adsorption–desorption cycle was finished. Triplicate tests were used to calculate the error bars.

### 2.7 X-ray absorption fine structure (EXAFS)

The U L_3_-edge X-ray absorption spectra were collected in transmission mode using an in-house laboratory-based X-ray absorption spectrometer. All XAFS data were analyzed using the program Demeter.^6^ For all samples, the EXAFS oscillations were extracted from the normalized XAS spectra by subtracting the atomic background using a quadratic spline fit to *k*^2^-weighted data, where *k* is the photoelectron wavenumber. The *χ*(*k*) functions were then Fourier-transformed into *R*-space. The Fourier-transform window was in the *k* range 2–10 Å^−1^. UO_2_(NO_3_)_2_·6H_2_O was used as a reference sample.

### 2.8 Computational method

All first-principles calculations were performed within the framework of density functional theory (DFT) as implemented in the plane wave set Vienna *Ab initio* Simulation Package (VASP) code. The exchange–correlation terms of the correlation functions were exchanged based on the generalized gradient approximation of Perdew–Burke–Ernzerhof (GGA–PBE). A periodically projected plane wave base set of the projector enhanced wave (PAW) was employed to calculate the electron–ion interaction. Spin polarization was considered in all simulations. Wave functions were expanded using a plane-wave basis set with kinetic energy cutoff of 500 eV. The convergence criterion of self-consistent iteration and the ion relaxation were set at 1 × 10^−4^ eV and 0.01 eV Å^−1^ to ensure the geometric configuration was sufficiently relaxed. A gamma *k*-point mesh of 3 × 3 × 3 for the Brillouin zone sampling was used for structural optimization. The above parameters were optimized until the energy change was negligible.

The formula for adsorption energy (Δ*G*_ads_) was defined as follows:Δ*G*_ads_ = *G*_MOF–UO_2_–*n*H_2_O_ − *G*_MOF_ − *G*_UO_2__ − *n* × *G*_H_2_O_where *G*_MOF_ and *G*_MOF–UO_2_–*n*H_2_O_ were the calculated total energy of pristine MOF or that loaded with UO_2_ and H_2_O guests. *G*_UO_2__ and *G*_H_2_O_ were defaulted as the energy of uranyl ions and water molecules. The *n* parameter represents the number of water molecules coordinated with a uranyl ion, ranging from 0 to 2.

## 3. Results and discussion

### 3.1 Synthesis and structure of ECUT-74

ECUT-74 was synthesized by the solvothermal method from self-assembly among Zn(NO_3_)_2_, 2-hydroxyterephthalic acid (H_3_TP), and 1,4-diazabicyclo[2.2.2]octane (DZO) in a mixed solution of dimethylacetamide (DMF) and ethylene glycol (EG) at 130 °C for two days. Although DZO is not contained in ECUT-74, its presence is vital for the synthesis of ECUT-74. The synthesis in detail is described in the Experimental section.

The structure of ECUT-74 was determined by single-crystal X-ray diffraction. This reveals a tetragonal crystal system with the space group of *P*4_1_2_1_2 (Table S1†). The asymmetric unit cell contains four crystallographically independent Zn sites, where Zn1 and Zn4 are of octahedral and pyramidal geometry, respectively, while Zn2 and Zn3 afford tetrahedral geometry (Fig. S1†). All these Zn sites are coordinated by oxygen atoms with Zn–O bond lengths of 1.935(6)–2.112(5) Å (Table S2†). The crystallographically independent ligands are composed of two completely deprotonated TP^3−^, one partially deprotonated H_2_TP^−^, one μ_3_-OH^−^ ion, and one DMF molecule. TP^3−^ takes μ_6_:η^1^η^1^η^1^η^2^η^2^ and μ_5_:η^1^η^1^η^1^η^1^η^2^ coordinated modes, while H_2_TP^−^ adopts the μ_2_:η^1^η^1^η^0^η^0^η^0^ coordinated mode (Fig. S2†). In light of this, the chemical formula of ECUT-74 is deduced to be Zn_4_(μ_3_-OH)(TP)_2_(H_2_TP)(DMF) in solvents.

**Fig. 1:**
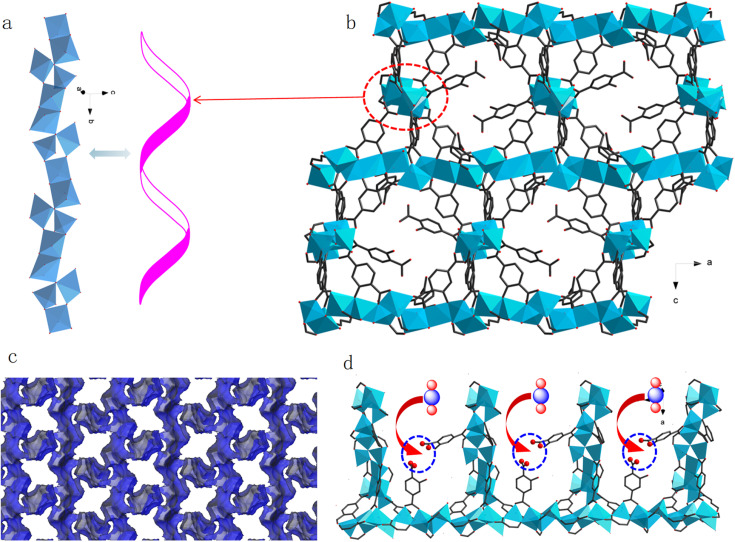
(a) View of the 1D helical rod-like building block along *b* direction. (b) View of the 3D framework built on helical rod-like building blocks along *b* direction. (c) View of the 3D channel in ECUT-74. (d) View of the charge-negative pockets each formed by two carboxyl groups from two adjacent helical rod-like building blocks.

Notably, these Zn sites are in turn combined together by μ_3_-OH^−^ ions and the hydroxyl groups to create a helical rod-like building block (Fig. 1a). In addition, these helical rod-like building blocks are further connected by TP^3−^ connectors to construct the final three-dimensional framework (Fig. 1b). Interestingly, we observe two outstanding structural advantages in ECUT-74 for driving uranium extraction from seawater. One is the three-dimensional channel in ECUT-74 (Fig. 1c), which is highly rare in MOFs based on the rod-like building block,^47^ as this category generally forms one-dimensional channels, such as in MOF-74,^48^ which is constructed from a ligand comparable to that used in ECUT-74. Such a three-dimensional nature theoretically endows ECUT-74 with a more rapid diffusion and then faster adsorption kinetics for the guest molecules within materials, such as uranium. The other advantage is the staggered stacking of the H_2_TP^−^-decorated helical rod-like building blocks, which forms a charge-negative pocket between two carboxyl groups from two adjacent helical chains with the four carboxyl oxygen atoms in a coplanar arrangement (Fig. 1d). Interestingly, such a charge-negative pocket can afford both the space and the geometry to perfectly match one plane-coordinated uranyl ion, thus theoretically endowing ECUT-74 with the precise uranyl-binding motif and then allowing selective uranium adsorption.

### 3.2 Robust nature of ECUT-74

**Fig. 2:**
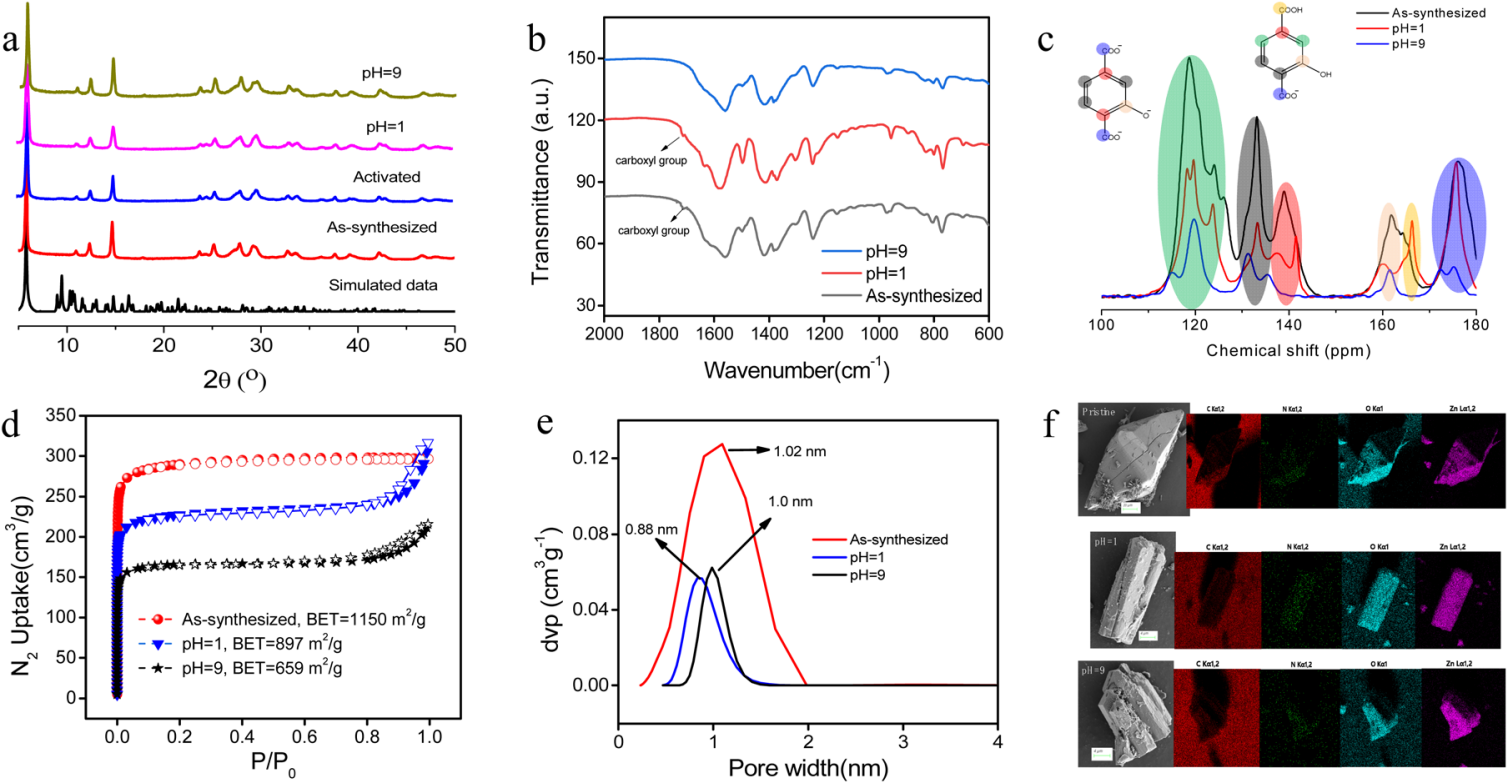
A comparison of (a) PXRD patterns, (b) IR spectra, (c) solid-state ^13^C MAS NMR spectra, (d) N_2_ adsorption–desorption isotherms, (e) pore size distribution, and (f) SEM-EDS for the simulated data, the as-synthesized samples, the samples after soaking in CH_3_OH and then activating under vacuum at 100 °C, the samples after soaking in water of pH = 1 for 24 h, and the samples after soaking in water of pH = 9 for 24 h.

Thermal stability of ECUT-74 was investigated by thermogravimetric analysis (TG), where the first major weight loss before 200 °C is due to removal of the solvent molecules and the decomposition temperature of the material is about 370 °C (Fig. S3†). The permanent porosity was disclosed by N_2_ adsorption–desorption isotherms at 77 K, giving BET surface areas of 1150 m^2^ g^−1^ with a narrow pore size of 1.02 nm (Fig. S4†). The chemical stability of ECUT-74 was investigated by soaking samples of ECUT-74 in water at pH values of 1 and 9, each for 24 hours and traced by powder X-ray diffraction (PXRD; Fig. 2a), IR spectra (Fig. 2b), solid-state ^13^C MAS NMR (nuclear magnetic resonance; Fig. 2c), N_2_ adsorption (Fig. 2d and e), and SEM-EDS (scanning electron microscopy plus energy dispersive spectrometer; Fig. 2f). Seen from a comparison in PXRD, IR, and solid-state ^13^C MAS NMR data, the retention of the MOF skeleton is confirmed after soaking ECUT-74 in water at pH of 1 and 9 for 24 h. In IR spectra, the free-standing carboxyl group in ECUT-74 is observed from the IR peak at 1710 cm^−1^, which is maintained at pH = 1, but disappeared at pH = 9, due to deprotonation on the carboxyl group at pH = 9. This can be confirmed from solid-state ^13^C MAS NMR, where at pH = 9 the peak of the carbon atom in COOH has disappeared, due to deprotonation. In N_2_ adsorption–desorption isotherms, the decrease in the N_2_ adsorption capacity and BET surface areas is due to a slight decrease in the crystal quality after soaking the samples in water at pH of 1 and 9, each for 24 h. However, the pore size is maintained. In SEM-EDS, some change in the morphology is observed after soaking the samples in water at pH of 1 and 9 for 24 h, but a single block without powderization is observed, while the basic elements in them show a uniform distribution in EDS images.

### 3.3 Antibiofouling activity of ECUT-74

**Fig. 3:**
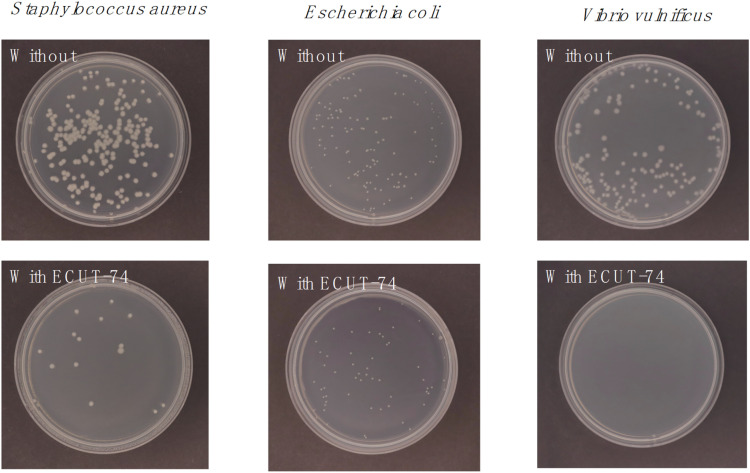
Antimicrobial activity of ECUT-74.

It has been observed that the presence of marine microorganisms in seawater would cause a reduction of uranium extraction performance of adsorbents, owing to marine biofouling.^32^ To investigate the antibiofouling activity of ECUT-74, we initially evaluated the inhibition rate of ECUT-74 for the G^+^ strain *Staphylococcus aureus* and the G^−^ strain *Escherichia coli*. It was found that the presence of ECUT-74 can effectively prevent the growth of these strains, giving inhibition rates of 91.9% and 54.6% (Fig. 3), respectively. Subsequently, we evaluated the inhibition rate of ECUT-74 for the marine strain *Vibrio vulnificus*, and a high inhibition rate of 100% was observed (Fig. 3). Clearly, ECTU-74 exhibits a good antibiofouling activity to support its application in uranium extraction from natural seawater.

### 3.4 Uranium extraction with ECUT-74

**Fig. 4:**
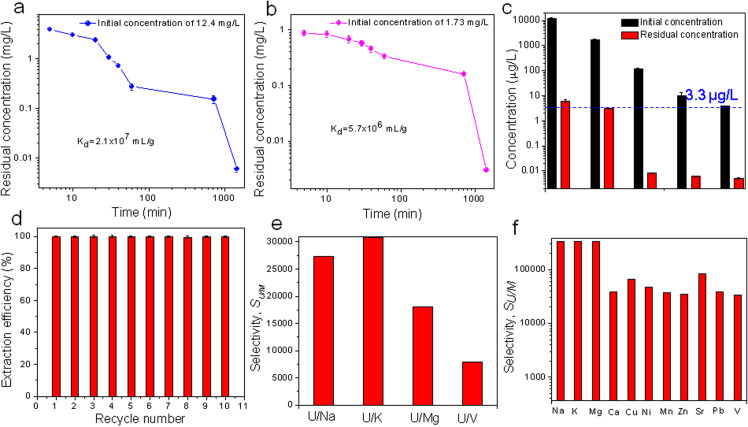
(a) Adsorption kinetics using a 12.4 mg L^−1^ uranyl solution. (b) Adsorption kinetics using a 1.73 mg L^−1^ uranyl solution. (c) A comparison of extraction ability by ECUT-74 for uranyl solutions with various concentrations. (d) Recycle tests of uranium extraction for a 10 mg L^−1^ uranyl solution with ECUT-74. (e) Uranium selectivity over Na, Mg, K, and V with 1 : 100 ratio in concentration from a binary mixture. (f) Uranium selectivity over 11 other ions with 1 : 1 ratio in concentration from a 12-ion mixture.

The adsorption kinetics of ECUT-74 was explored from a 50 mL 12.4 mg L^−1^ uranyl solution at pH = 5. Adsorption equilibrium is reached within 60 min, giving a 98% extraction efficiency, suggesting fast adsorption kinetics for ECUT-74. The residual uranium concentration after 24 h is 6 μg L^−1^ (Fig. 4a), suggesting an ultrahigh affinity of ECUT-74 towards uranyl ions. The corresponding *K*_d_ value, used to reflect such affinity, is calculated as up to 2.1 × 10^7^ mL g^−1^ (Fig. 4a), exceeding that of most reported materials for such use (Table S3†). The data can be well fitted by the pseudo-second-order kinetic model (Fig. S5, and Table S4†), suggesting a chemisorption by ECUT-74.^44^ The fast adsorption kinetics can be further confirmed using other uranium solutions with a lower uranium concentration such as 1.73 mg L^−1^ and 119.0, 10.0, and 3.86 μg L^−1^. For an initial concentration of 1.73 mg L^−1^, it can be reduced by ECUT-74 down to 3 μg L^−1^ after 24 h with a corresponding *K*_d_ value of 5.7 × 10^6^ mL g^−1^ (Fig. 4b). For other initial concentrations of 119.0, 10.0, and 3.86 μg L^−1^, the residual uranium concentration after 24 h is less than 0.01 μg L^−1^ (Fig. 4c), far below than the average uranium concentration (3.3 μg L^−1^) in seawater, strongly suggesting the potential of ECUT-74 for uranium extraction from seawater. Moreover, the effect of adsorbent dosage on uranium extraction was explored from a 50 mL 1.73 mg L^−1^ uranium solution at pH = 5 for 24 h (Fig. S6†). It was observed that the adsorbent dosage varying from 3 mg to 10 mg has little effect on the extraction efficiency, as evidenced by an extraction efficiency of more than 99.9% for all these adsorbent dosages. The adsorption isotherm was investigated using a 50 mL uranium solution with initial concentration varying from 12.4 mg L^−1^ to 208.6 mg L^−1^ at pH = 5 for 24 h, giving an uptake of 231 mg g^−1^ (Fig. S7†). The data can be well fitted by the Langmuir adsorption model (Fig. S7 and Table S5†), suggesting a monolayer adsorption on ECUT-74.^44^ The desorption and recovery of adsorbent were accomplished using 0.1 M HNO_3_ as an eluent, giving 99% desorption efficiency. Repeating this adsorption–desorption experiment ten times does not lead to any clear decrease in the uranium extraction performance (Fig. 4d), indicative of the good recyclability of ECUT-74. In addition, we also explored the pH effect on the uranium extraction from a 50 mL 1.73 mg L^−1^ uranium solution with pH from 2 to 10. It is found that at pH = 2 and 3, a relatively low extraction efficiency of less than 60% is observed, while a high extraction efficiency of >99% is observed at pH = 5–9 (Fig. S8†), confirming the applicability of ECUT-74 for uranium extraction from seawater with pH ∼8.

### 3.5 Selective adsorption of uranium

We next investigated the adsorption selectivity towards uranium over other ions from a binary mixed solution containing 1 mg L^−1^ uranyl ions and 100 mg L^−1^ other ions (Na, K, Mg, V) at pH = 8. Notably, even the presence of 100-times greater amount of other ions does not affect the uranium adsorption at all, as evidenced by the high extraction efficiency of more than 99% for U and less than 2% for Na, K, Mg, and V (Fig. S9†), as well as a uranium selectivity (*S*_U/M_, M = Na, K, Mg, and V) of 7.8 × 10^3^–3.0 × 10^4^ (Fig. 4e). Clearly, ECUT-74 offers an ultrahigh uranium selectivity over Na, K, Mg, and V. Especially, such *S*_U/V_ selectivity far exceeds that of all established materials for such use (Table S6†). In addition, we also explored the selective adsorption from a 12-ion mixed solution (Na, K, Mg, Ca, Cu, Ni, Mn, Zn, Sr, Pb, V, and U) with an initial concentration of 1 mg L^−1^ for each ion at pH = 8. Notably, the extraction efficiency towards uranium reaches 99.9%, while other ions give less than 3% extraction efficiency (Fig. S10†), also confirming the selective adsorption towards uranium over other cations. The corresponding uranium selectivity (*S*_U/M_, M = Na, K, Mg, Ca, Cu, Ni, Mn, Zn, Sr, Pb, V) is as high as 3.3 × 10^4^–3.3 × 10^5^ (Fig. 4f). These results suggest ECUT-74 as a good alternative for UES.

### 3.6 Extraction of uranium from seawater

The above results reveal a robust nature, a unique pocket-containing structure, and an outstanding adsorption performance towards UO_2_^2+^, indicative of a significant potential of ECUT-74 for UES. However, uranium exists as a composite anion, [UO_2_(CO_3_)_3_]^4−^, in seawater (pH value of ∼8). Theoretically, the capture of UO_2_^2+^ from such a [UO_2_(CO_3_)_3_]^4−^ composite anion can be achieved by a replacement reaction through exchanging the coordination inner boundary of CO_3_^2−^ by functionalized units from materials.^7,9,10^ In the literature, these functionalized units such as carboxylate and amidoxime have been found to be highly effective for such use,^19–29^ where the coordination between the functionalized units such as carboxylate and amidoxime and UO_2_^2+^, due to a replacement reaction, has been well realized to drive the uranium extraction from seawater. In this regard, it is suggested that the pocket formed by two carboxyl groups in ECUT-74 could be a good alternative for uranium extraction from seawater. To confirm this, we first tested the surface charge of ECUT-74 by means of zeta potential at pH = 2–10 (Fig. S11†). A positively charged surface was observed at pH = 2 and 3, while a negatively charged surface was observed at pH = 4–10. The positively charged surface could result from the protonation on the dimethylamine fragment of the coordinated DMF unit, while the negatively charged surface could stem from the deprotonation of the pocket on the carboxyl groups. Especially, the surface charge of ECUT-74 at pH = 8 is about −13.1 mV, implying the potential to drive strong electrostatic interactions, which will be conducive to UO_2_^2+^ capture.

**Fig. 5:**
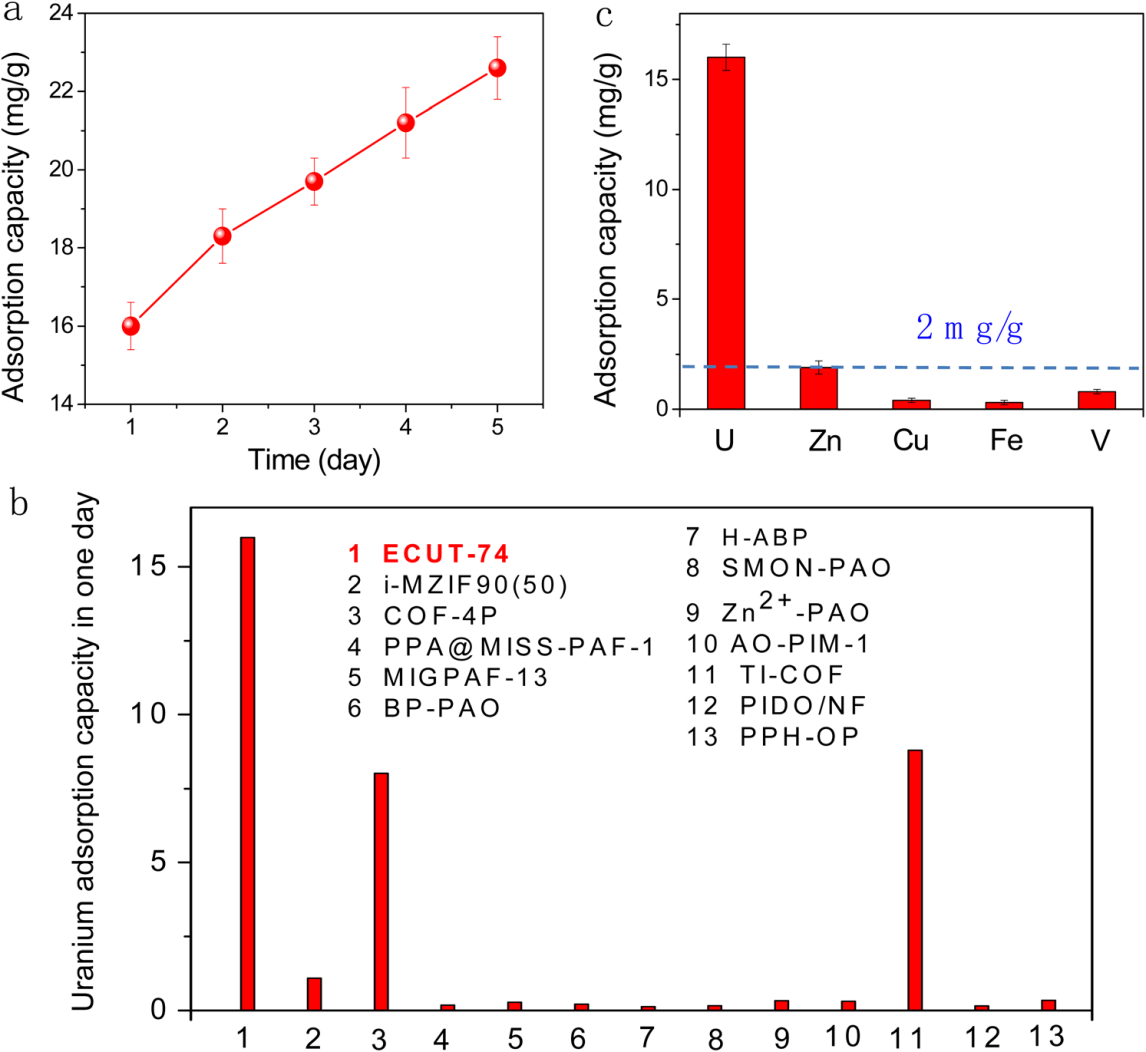
(a) Uranium adsorption capacity from seawater using ECUT-74 within 1–5 days. (b) A comparison of uranium adsorption capacity in one day among reported materials and our material. (c) A comparison of uranium adsorption capacity and other competing cations during uranium extraction from seawater.

Next, we explored the practical application for UES, for which 100 L of seawater with a uranium concentration of 3.3 μg L^−1^ was used. As shown in Fig. 5a, the uranium can be rapidly captured down to 1.7 μg L^−1^ by ECUT-74 in one day with 16 mg g^−1^ adsorption capacity. Extending the extraction time to five days will further reduce the uranium concentration down to 1.04 μg L^−1^, equal to an adsorption capacity of 22.6 mg g^−1^. A comparison in the adsorption ability in one day among established materials and ECUT-74 is illustrated in Fig. 5b and Table S7,† where our value far exceeds that of all reported materials, including benchmarks, such as i-MZIF90(50),^35^ PPH-OP,^36^ AO-PIM-1 (ref. 37) and MIGPAF-13.^40^

For most reported materials, they can efficiently capture both uranium and other competing metal ions, such as V, Cu, Fe, Zn from seawater,^18–40^ due to the inherent coordination competition. This will not only largely reduce the extraction ability of such materials, but also seriously block the separation of uranium from seawater. By contrast, this challenging issue can be solved using ECUT-74. As shown in Fig. 5c, ECUT-74 exhibits a high uranium adsorption capacity of 16 mg g^−1^ in one day, but an extremely low adsorption capacity of less than 2 mg g^−1^ for other metal ions, such as V, Cu, Fe, Zn. This means that ECUT-74 barely captures these competing metal ions, but does capture uranium. Using 0.1 M HNO_3_ as an eluent, we can conveniently implement the regeneration of the adsorbent, and no significant decrease in uranium extraction performance per day is observed after five cycles (Fig. S12†). Moreover, the cost of the material also represents a highly important factor for UES, from an economic perspective. Generally, the main cost of the current adsorbent is the cost of raw material for the synthesis of ECUT-74. Accordingly, the synthetic cost is estimated to be 1.66 $ per g (Table S8†), which is comparable to that of benchmark material COF-4 (2.7 $ per g) for such use,^31^ suggesting the economic feasibility of ECUT-74 for UES.

### 3.7 Uranium extraction mechanism for ECUT-74

To understand the uranium extraction mechanism for ECUT-74, we carried out a series of characterizations involving PXRD, SEM-EDS, N_2_ adsorption–desorption isotherms and XPS. The result from PXRD reveals the retention of the MOF framework even after the recycling after uranium extraction (Fig. S13†). The extraction of uranium using ECUT-74 can be intuitively reflected from SEM-EDS tests, where uranium element is found to show a uniform distribution on the material (Fig. S14†). N_2_ adsorption–desorption isotherms revealed a porosity with a BET value of 586 m^2^ g^−1^ after recycling following uranium extraction (Fig. S15†), which is comparable with that observed for the samples soaked in pH = 9 solution for 24 h. As shown in Fig. S16,† the XPS signal of U 4f can be split into two peaks at 392.1 eV (U 4f_5/2_) and 381.3 eV (U 4f_7/2_), which is comparable to the values observed in the literature, attributed to the +6 valence state of U(vi).^18^ Such values are smaller than that from UO_2_(NO_3_)_2_·6H_2_O (393.4 eV), suggesting a strong coordination interaction between the uranium species and ECUT-74. The extraction of uranium by ECUT-74 also shifts the O_1s_ binding energy towards a lower value (Fig. S17†), confirming the strong coordination interaction between the uranium species and ECUT-74.

**Fig. 6:**
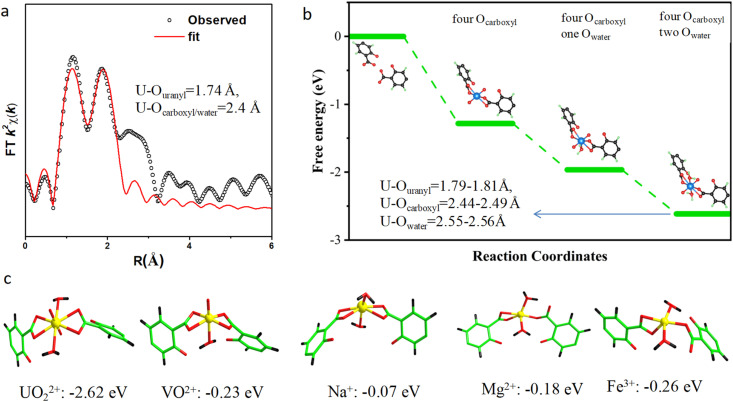
(a) Experimental EXAFS data (red) and fitting results (black). (b) View of the coordination environment of uranium in ECUT-74 calculated by DFT. (c) View of the coordination environment of uranium and other ions in ECUT-74 calculated by DFT, along with the binding energies.

Next, we carried out the measurement of U K-edge X-ray absorption near-edge structure (Fig. S18†) and EXAFS (Fig. S19†) spectroscopy to reveal the local coordination environment of the uranyl ion within ECUT-74. The result from EXAFS reveals the eight-coordination for the captured uranyl ion, composed of two uranyl oxygen atoms in axial position with U–O bond lengths of 1.74 ± 0.02 Å and six oxygen atoms on the equatorial plane with U–O bond lengths of 2.40 ± 0.02 Å (Fig. 6a and Table S9†).

Subsequently, we employed DFT calculations to reveal the exact coordination environment of the captured uranyl ion. It is found that the eight-coordination environment of the uranyl ion within ECUT-74 is the most stable (Fig. 6b), as evidenced by its lowest adsorption energy (−2.62 eV). The coordination atoms are composed of two inherent uranyl oxygen atoms, four carboxyl oxygen atoms from two adjacent helical rod building blocks, and two coordinated water molecules. The four carboxyl oxygen atoms and the two coordinated water molecules are located in the same plane. The U–O_uranyl,_ U–O_carboxyl_, and U–O_water_ bond lengths are 1.79–1.81 Å, 2.44–2.49 Å, and 2.55–2.56 Å, respectively. These values are in good agreement with the results from EXAFS, and it is certain that the coordination fixing by four carboxylate oxygen atoms from two adjacent helical rod building blocks should be responsible for the current uranyl extraction in ECUT-74. Moreover, we also carried out DFT calculations for the adsorption of other representative ions, such as Na^+^, Mg^2+^, Fe^3+^, and VO^2+^ within this pocket (Fig. 6c). The adsorption structures of them are shown in Fig. 6c. Seen from the binding energy, it is clear that such a pocket in ECUT-74 affords a superior affinity towards UO_2_^2+^, far exceeding that towards other representative ions such as Na^+^, Mg^2+^, Fe^3+^, and VO^2+^, implying a high selectivity towards UO_2_^2+^ over other ions. This is in good agreement with the experimental results.

## 4. Conclusion

In summary, we demonstrated a robust biomimetic MOF, ECUT-74, featuring a novel helical-rods-in-staggered-array framework with embedded three-dimensional ion-conduction channels and negatively charged uranyl-binding pockets. This hierarchical architecture enables superior uranium extraction performance through three synergistic mechanisms: (i) precision binding-site engineering with a strong uranyl-fixing ability (*K*_d_ = 2.1 × 10^7^ mL g^−1^) and steric exclusion effects against competing cations (*S*_U/V_ = 10^4^); (ii) dual drive for promoting uranium capture with one being the local acidity of the carboxyl groups that leads to the decomposition of [UO_2_(CO_3_)_3_]^4−^ to UO_2_^2+^ ions, the other one being the deprotonation of carboxyl groups that leads to the negative charge of this pocket for Coulomb interaction with uranyl ions; (iii) the location of the uranyl-binding pocket in the three-dimensional channel that accelerates the mass transfer of uranium within the material. The practical application in seawater reveals a fast and efficient uranium extraction with a 16 mg g^−1^ uptake capacity in just one day, along with high selectivity and good reusability, in conjunction with a low synthetic cost, confirming its superior application prospects in uranium recovery from seawater.

## Author contributions

A. N. Y., Y. X. L., X. Q. X.: methodology, formal analysis, investigation, writing, validation. L. L. G.: theoretical calculation. F. L.: conceptualisation, writing – review & editing, supervision, funding acquisition, resources.

## Conflicts of interest

There are no conflicts to declare.

## Supplementary Material

SC-016-D5SC02966J-s001

## Data Availability

The data supporting this article have been included as part of the ESI.†
