# Correction: Robust biomimetic MOF featuring a negative pocket for precise recognition of uranyl, enabling ultrahigh U/V selectivity and rapid uranium extraction from seawater

**DOI:** 10.1039/d6sc90189a

**Published:** 2026-10-05

**Authors:** Anni Ye, Yuxuan Liu, Lele Gong, Xianqing Xie, Feng Luo

**Affiliations:** a National Key Laboratory of Uranium Resources Exploration-Mining and Nuclear Remote Sensing, East China University of Technology Nanchang 330013 China ecitluofeng@163.com; b State Key Laboratory of NBC Protection for Civilian Beijing 100191 China; c National Engineering Research Center for Carbonhydrate Synthesis, Jiangxi Normal University Nanchang 330027 China

## Abstract

Correction for ‘Robust biomimetic MOF featuring a negative pocket for precise recognition of uranyl, enabling ultrahigh U/V selectivity and rapid uranium extraction from seawater’ by Anni Ye *et al.*, *Chem. Sci*., 2025, **16**, 13749–13759, https://doi.org/10.1039/d5sc02966j.

The authors apologise for the following errors present in the main manuscript and Supporting Information (SI).

In eqn (2), there is an additional ×10^3^ present, which should be omitted. The corrected eqn (2) is shown below.2
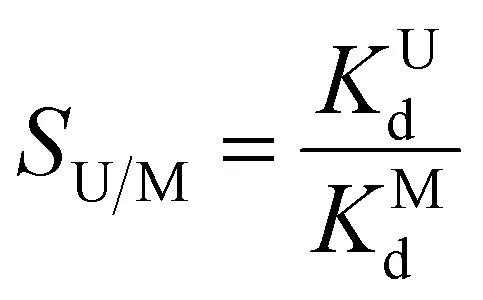


The calculation of the cost of Zn(NO_3_)_2_ in Table S8 is incorrect, and should be 0.22 $ per g, rather than the current 0.02 $ per g. As a result, the corresponding total cost should be 1.86 $ per g, rather than the current 1.66 $ per g, as also detailed on page 13757 of the main manuscript.

In Fig. S7, the fitting of the Langmuir data does not use the first data point, which was not noted in the figure.

The SI of the original article has been updated with the corrected version.

The Royal Society of Chemistry apologises for these errors and any consequent inconvenience to authors and readers.

