# Supplementary material for: Robust biomimetic MOF featuring a negative pocket for precise recognition of uranyl, enabling ultrahigh U/V selectivity and rapid uranium extraction from seawater
Source: Chem Sci. 2025 Jun 20;16(30):13749–59. doi: 10.1039/d5sc02966j (PMC12210057; doi:10.1039/d5sc02966j)
Supplement: SC-016-D5SC02966J-s001 [file SC-016-D5SC02966J-s001.pdf]

Supplementary Information (SI) for Chemical Science.
This journal is © The Royal Society of Chemistry 2025

Robust biomimetic MOF featuring a negative pocket for precise recognition of uranyl enabling an ultrahigh U/V selectivity and a rapid uranium extraction from seawater

Anni Ye,[a,‡] Yuxuan Liu,[a, ‡] Lele Gong,[b, ‡] Xianqing Xie,[c] and Feng Luo[a*]

[a]National Key Laboratory of Uranium Resources Exploration-Mining and Nuclear Remote Sensing,, East China University of Technology, Nanchang 330013, China, e-mail: ecitluofeng@163.com

[b]State Key Laboratory of NBC Protection for Civilian, Beijing 100191, China

[c]National Engineering Research Center for Carbonhydrate Synthesis, Jiangxi Normal University, Nanchang, 330027, China

[‡]These authors contributed equally: Anni Ye, Yuxuan Liu, and Lele Gong

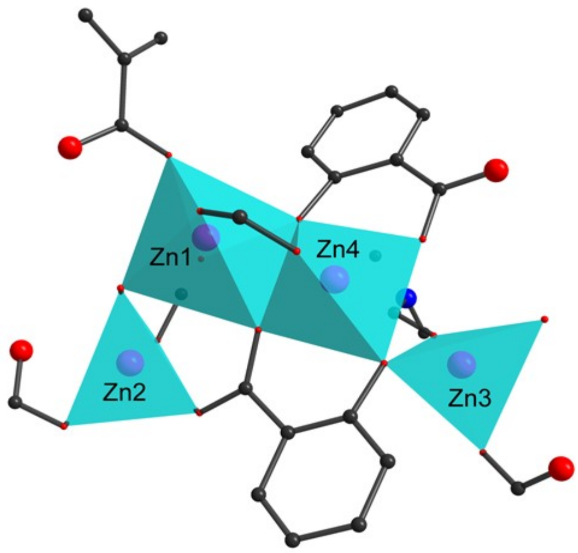

Fig. S1 View of the coordination surrounding around Zn sites.

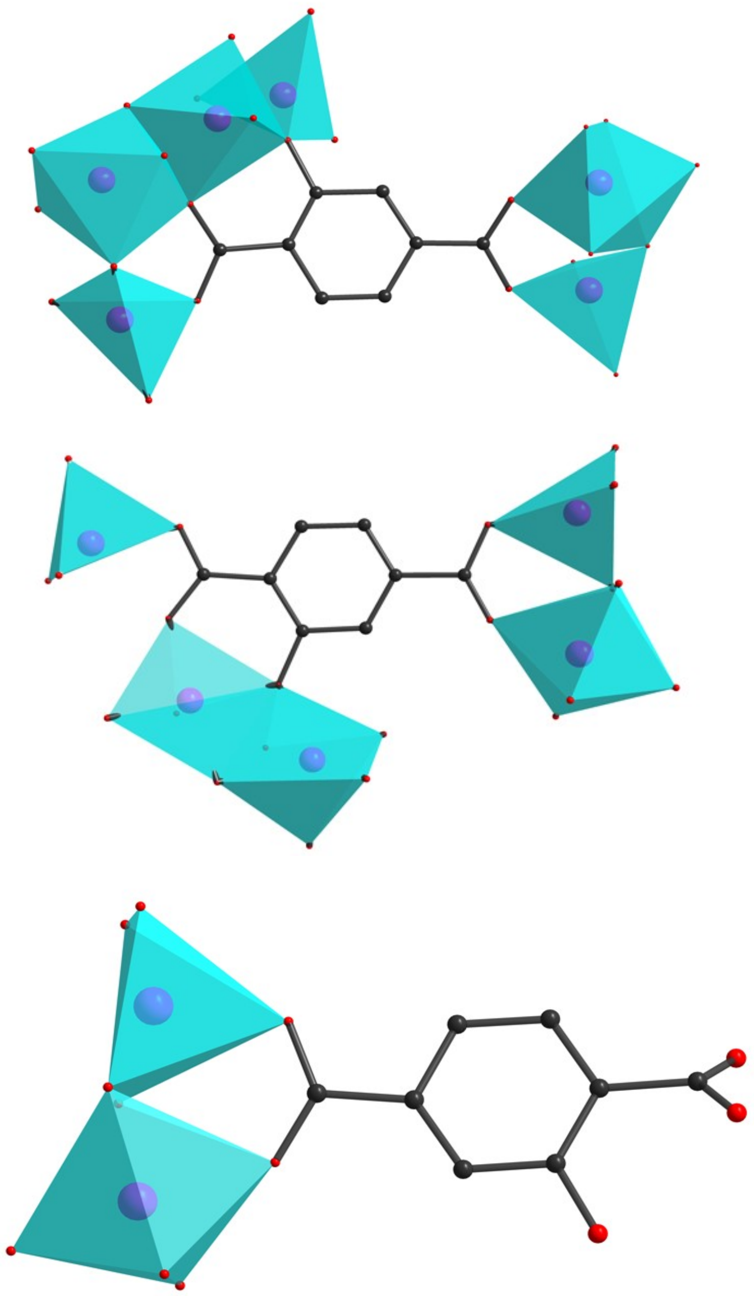

Fig. S2 View of the coodination modes of TP^{3-} and H_2TP^{-} ligands in μ_6:$\eta^1\eta^1\eta^1\eta^2\eta^2$, μ_5:$\eta^1\eta^1\eta^1\eta^1\eta^2$, and μ_2:$\eta^1\eta^1\eta^0\eta^0\eta^0$, respectively.

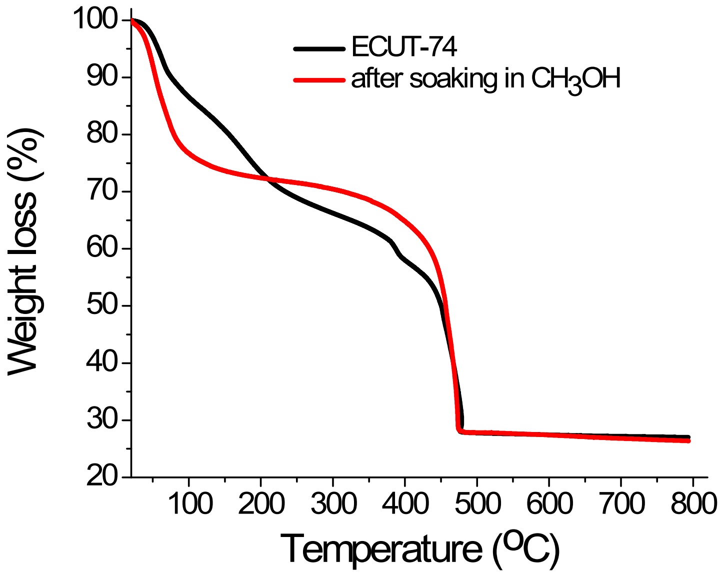

Fig. S3 TG plots of ECUT-74 and the samples of ECUT-74 after soaking CH_3OH for three days. This data was measured in air.

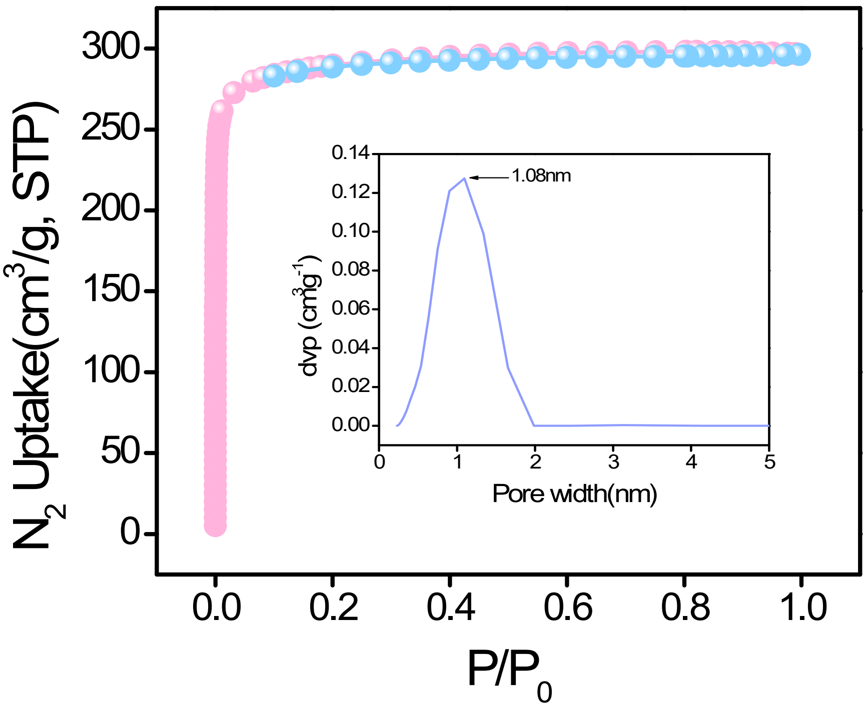

Fig. S4 N_2 adsorption and desorption isotherms of ECUT-74 at 77 K with the inset of aperture distribution.

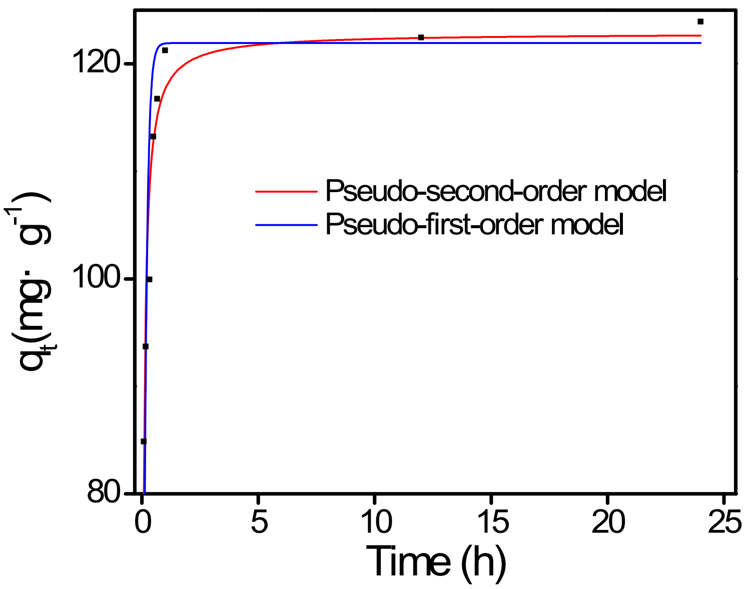

Fig. S5 A fitting the data of the adsorption kinetics from the 12.4 mg/L uranyl solution by the pseudo-first-order kinetic models and the pseudo-second-order kinetic models, respectively.

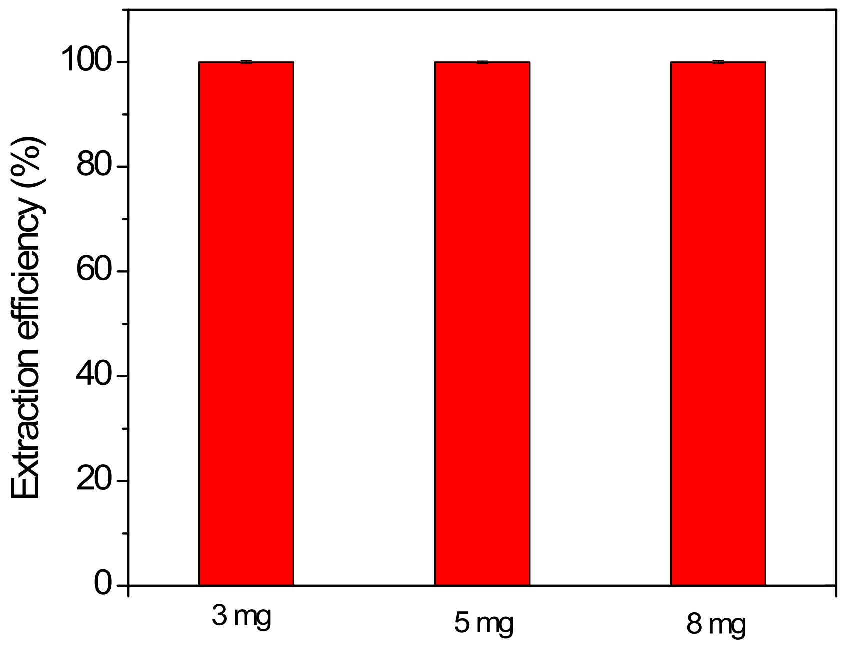

Fig. S6 The effect of adsorbent dosage on the uranium extraction.

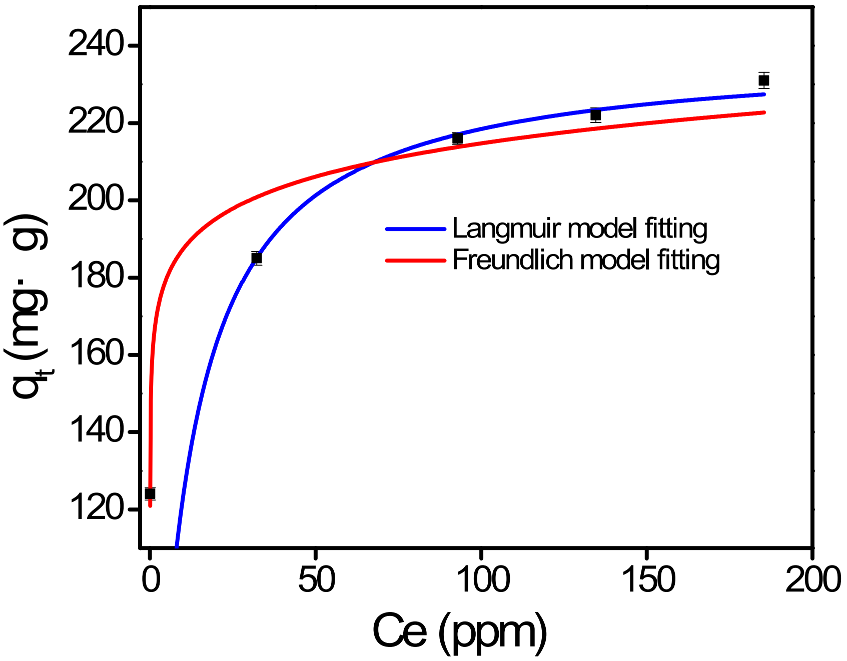

Fig. S7 A fitting the data of the adsorption isotherm by the Langmuir and Freundlich models, respectively.

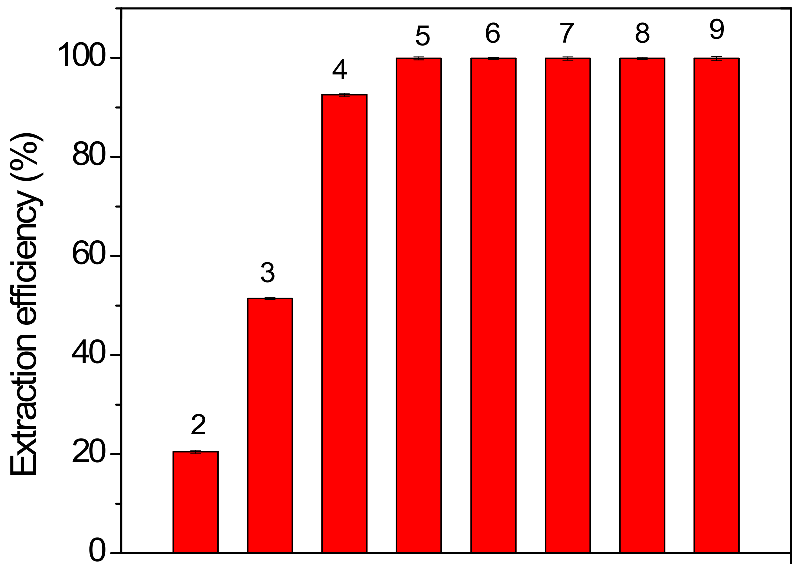

Fig. S8 The effect of pH value on the uranium extraction.

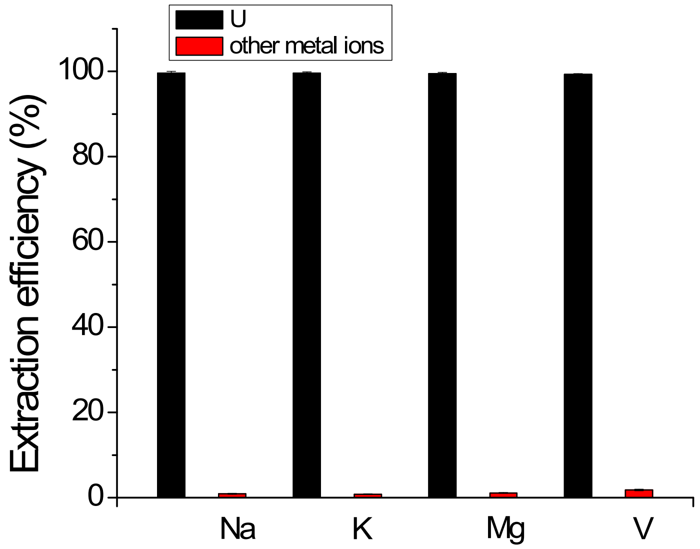

Fig. S9 Extraction efficiency of uranium and other ions from a binary mixed solution containing 1 mg/L uranyl ions and 100 mg/L other ions (Na, K, Mg, V).

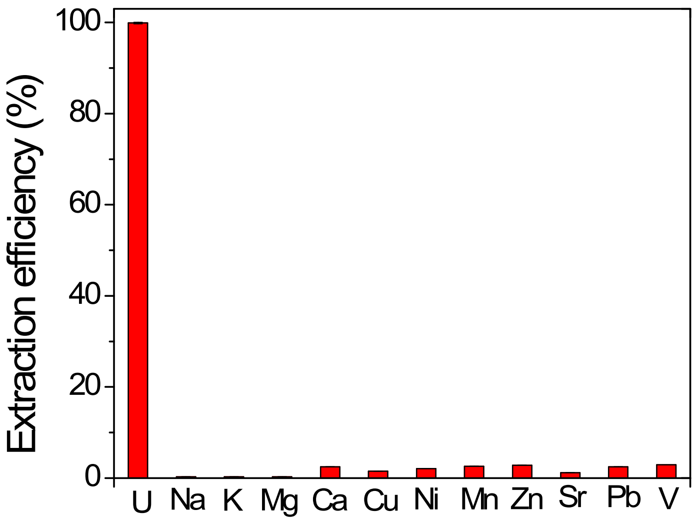

Fig. S10 Extraction efficiency of uranium and other ions from a 12-ions mixed solution containing 1 mg/L uranyl ions and 1 mg/L other ions (Na, K, Mg, Ca, Cu, Ni, Mn, Zn, Sr, Pb, and V).

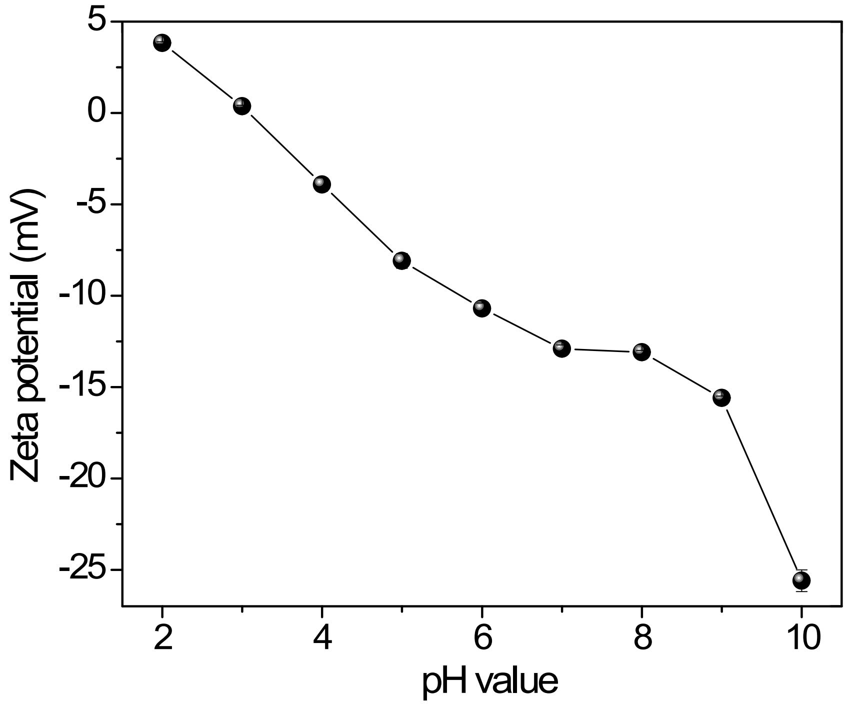

Fig. S11 Zeta potential of ECUT-74 under pH value of 2-10.

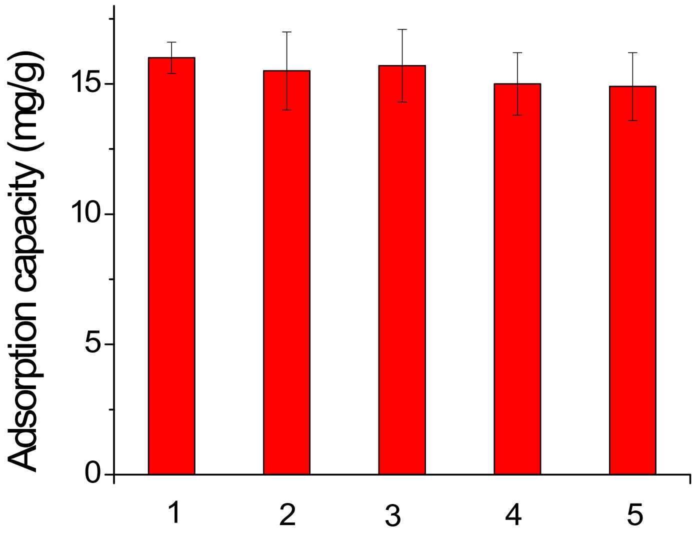

Fig. S12 Recycle tests of uranium extraction from seawater by ECUT-74. The extracting time is one day.

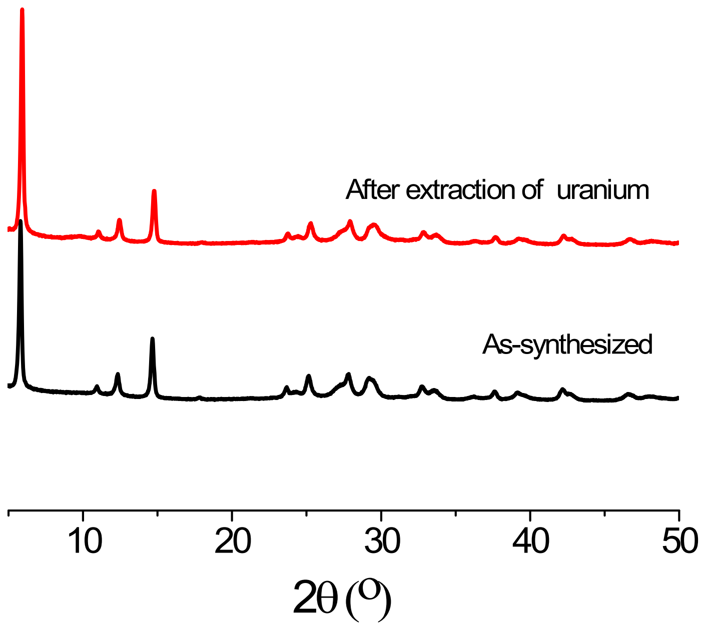

Fig. S13 A comparison of PXRD between the as-synthesized samples of ECUT-74 and the samples after the recycle of uranium extraction.

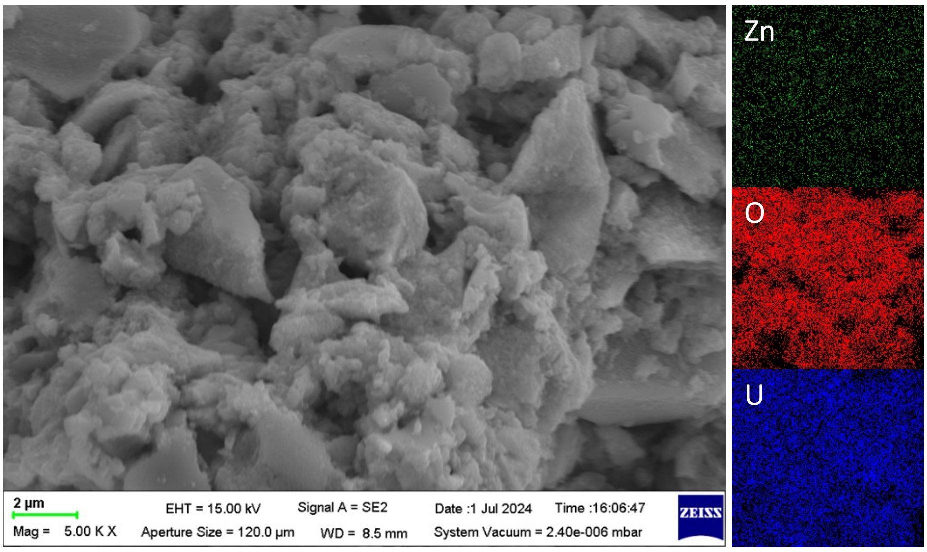

Fig. S14 SEM-EDS of the samples of ECUT-74 after extraction of uranium.

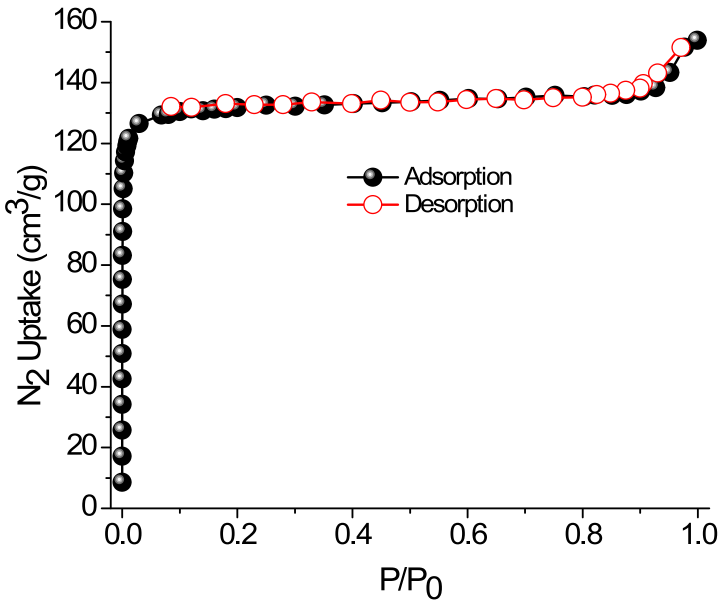

Fig. S15 N_2 adsorption-desorption isotherm after recycle of uranium extraction.

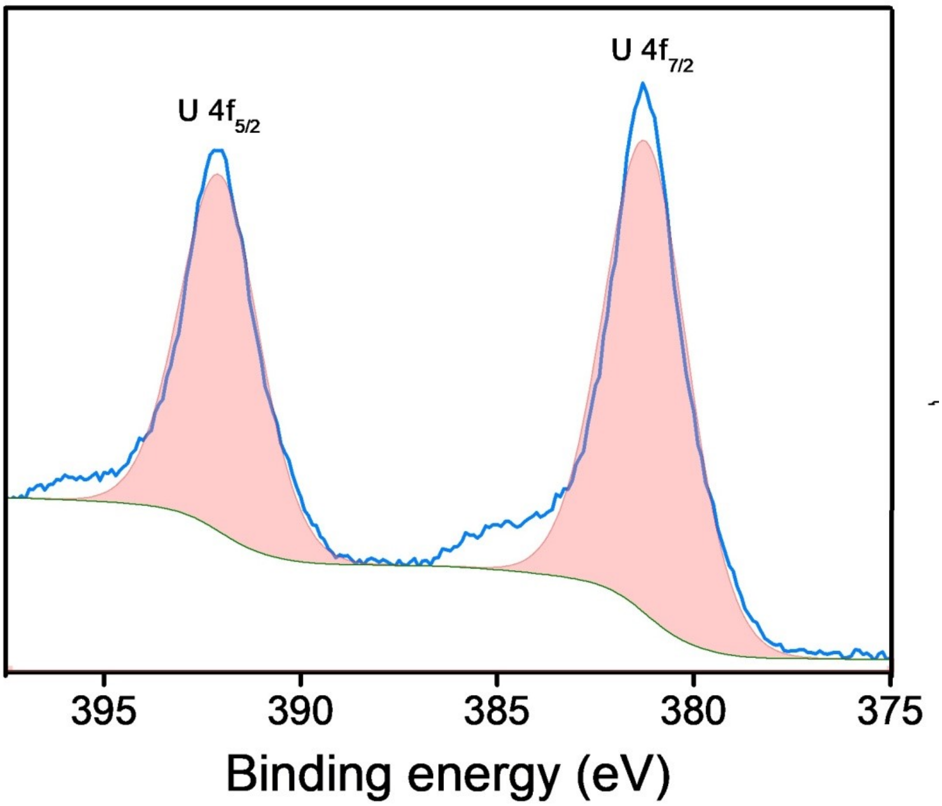

Fig. S16 XPS U element of the samples of ECUT-74 after extraction of uranium.

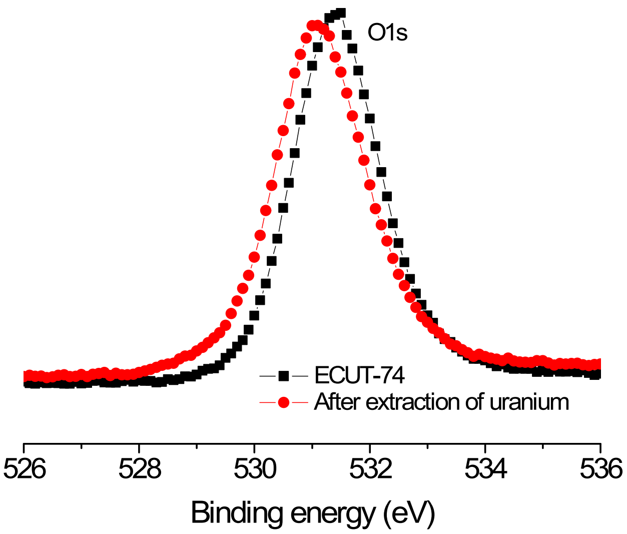

Fig. S17 XPS O element of the samples of ECUT-74 after extraction of uranium.

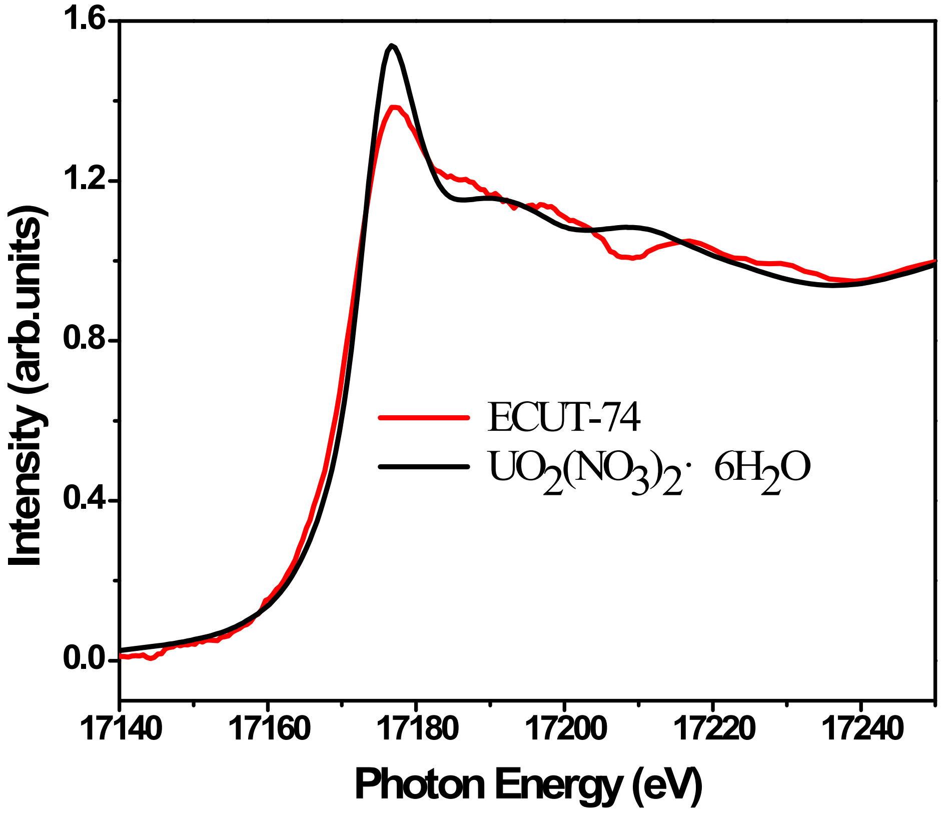

Fig. S18 A comparison in XANES between ECUT-74 after extraction of uranium and the $UO_2(NO_3)_2 \cdot 6H_2O$.

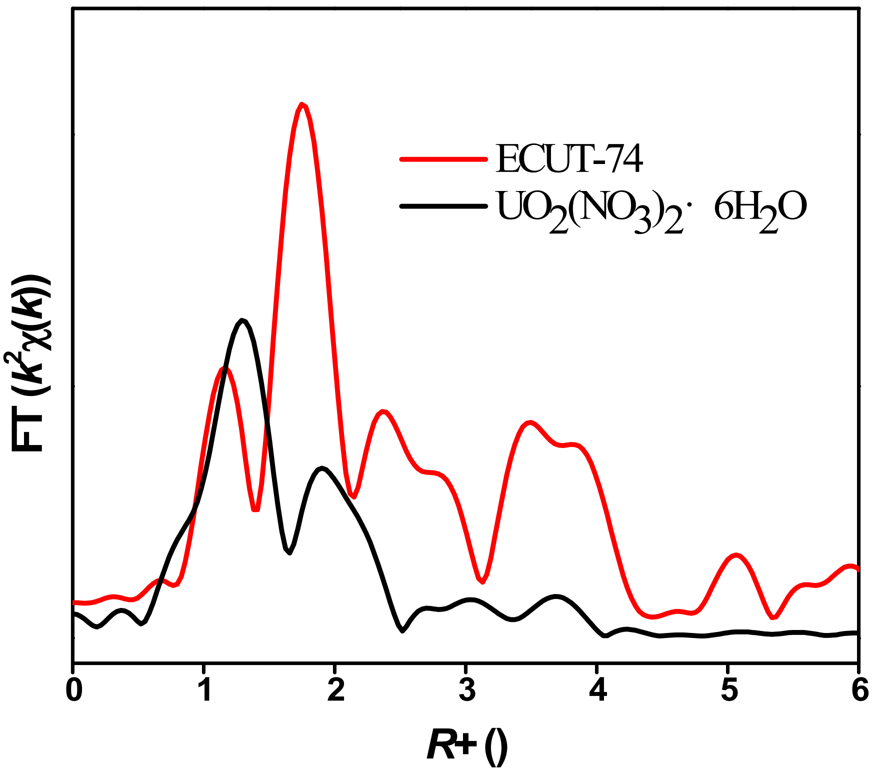

Fig. S19 A comparison in EXAFS between ECUT-74 after extraction of uranium and the $UO_2(NO_3)_2·6H_2O$.

Table S1. The crystal data of ECUT-74.

Temperature	303(2) K
Wavelength	0.71073 Å
Crystal system, space group	Tetragonal, P4(1)2(1)2
Unit cell dimensions	a=b=17.1567(4) Å<br>c=33.4185(11) Å
Volume	9836.8(5) $Å^3$
F(000)	3480
Completeness to theta=25.00	99.8 %
Goodness-of-fit on F^2	0.996
Final R indices [I>2sigma(I)]	R1=0.0699, wR2=0.1845
CCDC number	2373411

Table S2. Zn-O bond lengths in ECUT-74.

O(1)-Zn(4)	1.994(5)
O(1)-Zn(1)	2.004(6)
O(2)-Zn(4)	2.016(6)
O(2)-Zn(1)	2.362(6)
O(3)-Zn(3)	1.983(6)
O(3)-Zn(4)	2.019(6)
O(4)-Zn(2)	1.983(6)
O(5)-Zn(4)	1.954(6)
O(5)-Zn(2)#1	2.417(7)
O(6)-Zn(3)#2	1.935(6)
O(7)-Zn(1)#3	2.030(7)
O(8)-Zn(1)#4	2.040(6)
O(9)-Zn(2)#4	1.949(5)
O(10)-Zn(2)#1	1.978(6)
O(11)-Zn(1)	2.138(8)
O(12)-Zn(4)	2.024(9)
Zn(1)-O(16)#6	2.112(5)
Zn(2)-O(16)#6	1.946(5)
Zn(3)-O(16)	1.990(6)
Zn(3)-O(17)	2.024(12)

Table S3. A comparison of K_d value for uranium extraction among established adsorbents and our case.

Adsorbents	K_d value (mL/g)	References
ECUT-74	2.1×10^7	This work
MIGPAF-13	2.0×10^6	M1
SMON-PAO	3.76×10^5	M2
PIDO/NF	2.84×10^5	M3
MS@PIDO/Alg	1.98×10^4	M4
POP_1-AO	1.1×10^6	M5
PPA@MISS-PAF-1	2.18×10^7	M6
i-MZIF90(50)	1.22×10^7	M7

Table S4. Fitting the data of the adsorption kinetics from the 12.4 mg/L uranyl solution by the pseudo-first-order kinetic models and the pseudo-second-order kinetic models, respectively.

Pseudo-first-order model

q_e: adsorption capacities at equilibrium ($mg \cdot g^{-1}$)

$q_t = q_e(1 - e^{\frac{-k_1 t}{2.303}})$ q_t: adsorption capacities at time t ($mg \cdot g^{-1}$)

k_1: pseudo-first-order rate constant for the kinetic model (min^{-1})

Pseudo-second-order model

$q_t = (q_e^2 * k_2 t)/(1 + q_e k_2 t)$ k_2: pseudo-second-order rate constant of adsorption ($mg \cdot g^{-1} \cdot min^{-1}$)

	Pseudo-first-order				Pseudo-second-order		
Adsorbent	$q_{e,exp}(mg \cdot g^{-1})$	$q_{e,cal}(mg \cdot g^{-1})$	$K_1(min^{-1})$	R^2	$q_{e,cal}(mg \cdot g^{-1})$	$K_2(g \cdot mg^{-1} \cdot min^{-1})$	R^2
ECUT-74	123.98	121.93	18.424	0.89	124.64	0.1849	0.99

Table S5. Fitting the data of the adsorption isotherm by the Langmuir and Freundlich models, respectively.

**Langmuir model**	
$Q_e = K_L Q_{max} C_e/(1 + KC_e)$	Q_e: adsorption capacities at equilibrium (mg·g^{-1})<br>Q_{max}: Maximum adsorption capacity (mg·g^{-1})<br>K： Constants related to adsorption (mg·g-1)
**Freundlich model**	
$Q_e = K_F C_e^{-n}$	n: Parameters related to the intensity of adsorption

	Langmuir model				Freundlich model		
Adsorbent	$Q_{e,exp}$(mg·g^{-1})	K_L(L·mg^{-1})	Q_{max}(mg·g^{-1})	R^2	K_F (mg·g^{-1})	n	R^2
ECUT-74	231	0.10693	238.9	0.99	163.589	0.05908	0.95

Table S6. A comparison of U/V for uranium extraction among established adsorbents and our case.

Adsorbents	U/V selectivity	References
ECUT-74	3.3×10^{4}	This work
PPH-OP	1.0×10^{2}	M8
MIGPAF-13	60	M1
UiO-66-3C4N	17	M9
H-ABP	1.3	M10
Zn^{2+}-PAO	<1	M11
PAF-170-AO	15	M12
BP-PAO	<1	M13
POP_1-AO	<4	M5
AO-PIM-1	<1	M14

Table S7. A comparison of uranium extraction capacity from natural seawater among established adsorbents and our case.

Adsorbents	Uranium extraction capacity (mg/g)	Extraction time (day)	Uranium extraction capacity *per* day (mg/g/day)	References
ECUT-74	16	1	16	This work
i-MZIF90(50)	28.2	25	1.13	M7
PPA@MISS-PAF-1	16.97	90	0.19	M6
BP-PAO	11.76	56	0.21	M13
H-ABP	11.5	90	0.13	M10
SMON-PAO	9.59	56	0.17	M2
Zn^{2+}-PAO	9.23	28	0.33	M11
PIDO/NF	8.7	56	0.16	M3
COF-4P	24.06	3	8.02	M15
PPH-OP	7.12	21	0.34	M8
AO-PIM-1	9.03	28	0.32	M14
MIGPAF-13	16	56	0.28	M1
TI-COF	8.8	1	8.8	M16
MITpBD	23.6	7	3.37	M17
JNM-101-AO	7.96	8	1.0	M18

Table S8. The cost of the raw materials used to synthesize ECUT-74.

The cost of the raw materials from Aladdin Biochemical Technology Co., Ltd	The cost of the raw materials for the synthesis of ECUT-74
2-hydroxyterephthalic acid (1.5 \$/g)<br>$Zn(NO_3)_2\cdot 6H_2O$ (0.076 \$/g)<br>1,4-diazabicyclo[2.2.2]octane (0.04 \$/g)<br>DMF (6.88 \$/L)<br>glycol (6.56 \$/L)<br>methanol (4.88 \$/L)	2-hydroxyterephthalic acid (1.08 \$),<br>$Zn(NO_3)_2\cdot 6H_2O$ (0.02 \$)<br>1,4-diazabicyclo[2.2.2]octane (0.01 \$)<br>DMF (0.27 \$)<br>glycol (0.09 \$)<br>methanol (0.19 \$)
	Total: 1.66 \$/g

Table S9. Parameters of the fit of the samples of ECUT-74 after uranium extraction.

Pair	CN	R(Å)	$\sigma^2 \times 10^{-3}$ (Å^{-3})	R factor
U-O_1	2.0	1.74±0.02	1.0	0.011
U-O_2	5.5± 1.0	2.40±0.02	2.4	

References

J. Am. Chem. Soc. 2021, 143, 14523-14529/M1

Adv. Funct. Mater.2019, 29, 1805380/M2

Adv. Energy Mater. 2018, 8, 1802607/M3

Adv. Funct. Mater. 2019, 29, 1901009/M4

ACS Cent. Sci. 2021, 7, 1650-1656/M5

Chem 2020, 6, 1683-1691/M6

Energy Environ. Sci. 2022, 15, 3462-3469/M7

Nat. Sustain. 2021, 4, 708-714/M8

Angew. Chem., Int. Ed. 2020, 59, 4262-4268/M9

Energy Environ. Sci. 2019, 12, 1979-1988/M10

Adv. Mater. 2020, 32, 1906615/M11

Chem. Sci. 2020, 11, 4747-4752/M12

Angew. Chem., Int. Ed. 2020, 59, 1220-1227/M13

Nat. Sustain. 2022, 5, 71-80/M14

Angew. Chem., Int. Ed. 2023, 62, e202303129/M15

Chem. Sci. 2024, 15, 10882–10891/M16

Nat. Water 2025, 3, 89-98/M17

Sci. China Chem. 2025, 68, 1906-1915/M18
